# Do Aminopenicillins Have a Role in Treating Ampicillin-Resistant *Enterococcus* UTIs?

**DOI:** 10.1093/ofid/ofaf619

**Published:** 2025-10-08

**Authors:** Armani M Hawes, Pranita D Tamma, Navaneeth Narayanan

**Affiliations:** Division of Infectious Diseases, Department of Medicine, Johns Hopkins University School of Medicine, Baltimore, Maryland, USA; Department of Pediatrics, Johns Hopkins University School of Medicine, Baltimore, Maryland, USA; Department of Pharmacy Practice and Administration and Center of Excellence in Pharmaceutical Translational Research and Education, Rutgers University Ernest Mario School of Pharmacy, Piscataway, New Jersey, USA; Division of Infectious Diseases, Department of Medicine, Rutgers University Robert Wood Johnson Medical School, New Brunswick, New Jersey, USA

**Keywords:** aminopenicillins, antimicrobial resistance, bacterial infections, pharmacokinetics/pharmacodynamics, urinary tract infections

## Abstract

Urinary tract infections caused by *Enterococcus* species are a frequent clinical challenge. The rising prevalence of ampicillin resistance often seemingly precludes the use of aminopenicillins. However, evidence suggests that aminopenicillins may retain clinical efficacy, even when susceptibility testing indicates resistance. Integrating available pharmacokinetics/pharmacodynamics and clinical data and risk-benefit considerations, we seek to address the question: Can aminopenicillins still be a viable treatment option in the management of ampicillin-resistant *Enterococcus* infections?

A 72-year-old woman with a history of osteoarthritis, diabetes mellitus, and hypertension presents to her primary care provider with a 2-day history of dysuria, urinary urgency, and suprapubic discomfort. She appears relatively well. Her vital signs are within normal limits: she is afebrile, with a heart rate of 83 beats per minute, respiratory rate of 12 breaths per minute, and blood pressure of 142/84 mmHg. Physical examination reveals no costovertebral angle tenderness. Based on her localized urinary symptoms and absence of systemic findings, a diagnosis of uncomplicated cystitis is suspected.

A clean-catch urinalysis reveals 250 white blood cells per high-power field and no squamous epithelial cells, indicating a reliable sample. A urine culture is obtained, and while results are pending, the patient is empirically prescribed amoxicillin as she had grown a pan-susceptible *Escherichia coli* from her urine 3 months prior. By day 3, the culture grows >100 000 colony-forming units of *Enterococcus faecium*, resistant to ampicillin (a surrogate marker for amoxicillin susceptibility [1]). The isolate is susceptible to linezolid. The primary care provider contacts the patient with the intent to discontinue amoxicillin and initiate linezolid. However, during the conversation, the patient reports complete resolution of her urinary symptoms.

This hypothetical case illustrates a scenario not uncommon in outpatient practice. *Enterococcus* species are recognized pathogens in urinary tract infections (UTIs) [2], and ampicillin-resistant strains are increasingly encountered [3]. While amoxicillin is typically the treatment of choice for *E. faecalis* UTIs, it is generally not favored for empiric management of uncomplicated cystitis, which is most commonly caused by *E. coli* [2]. Notably, *E. coli* resistance to amoxicillin among uropathogens can range from 30% to 90%, depending on geographic location [4]. Agents such as nitrofurantoin or trimethoprim–sulfamethoxazole are generally preferred for empiric treatment of uncomplicated cystitis [5]. Nonetheless, this case highlights a scenario in which an ampicillin-resistant *Enterococcus*-associated cystitis resolved with amoxicillin monotherapy, obviating the need for linezolid. We suggest that under certain circumstances, aminopenicillins may still be clinically effective against *Enterococcus* species classified as resistant in vitro, a concept explored in the discussion below.

## SELECTING THE RIGHT DRUG AT THE RIGHT DOSE FOR THE RIGHT INDICATION

According to the Clinical and Laboratory Standards Institute (CLSI), *Enterococcus* species exhibiting a minimum inhibitory concentration (MIC) ≥ 16 µg/mL are classified as resistant to ampicillin [1]. MIC breakpoints for most organisms are established based on systemic (ie, plasma) drug concentrations. Cefazolin serves as a notable exception, with distinct breakpoints for gram-negative organisms isolated from the lower urinary tract (susceptible ≤16 µg/mL) versus nonurinary sources (susceptible ≤2 µg/mL) [1]. Nevertheless, antibiotic selection is informed not solely by plasma pharmacokinetic/pharmacodynamic (PK/PD) parameters but also by consideration of drug concentrations at the site of infection, in addition to clinical factors.

## CONSIDERATION OF ANTIBIOTIC CONCENTRATIONS IN THE URINE

While certain antimicrobial agents attain high systemic concentrations, they may not achieve therapeutic levels in the urine and are therefore suboptimal choices for UTIs. Moxifloxacin exemplifies this principle. A large fraction of moxifloxacin (>50%) is hepatically metabolized to inactive metabolites [6]; only ∼20% is eliminated unchanged in the urine which is a stark contrast to urinary drug concentrations observed with levofloxacin or ciprofloxacin. Therefore, moxifloxacin is not suggested for the treatment of UTIs. Conversely, some antibiotics concentrate extensively in the urine, despite limited systemic exposure. Nitrofurantoin is one such example. It does not achieve therapeutic serum levels but is present in the urine at concentrations of ∼200 µg/mL, well above typical nitrofurantoin MIC distributions, making it effective for the treatment of cystitis [7, 8].

Currently, urinary breakpoints for *Enterococcus* species have not been established. Nonetheless, PK data support the use of aminopenicillins for the treatment of enterococcal UTIs; aminopenicillins achieve urinary concentrations that exceed the MICs commonly observed in clinical isolates. Two PK studies demonstrated mean 0–6 hour urine concentrations of 482–580 µg/mL and 1100–1579 µg/mL following administration of 250 and 500 mg, respectively, of amoxicillin [9, 10]. Sub-MIC antimicrobial exposure slows bacterial growth, explaining why urine antimicrobials can be effective even when urinary bladder drug levels are below the MIC [11, 12]. Notably, amoxicillin achieves higher levels in the urine compared to ampicillin, potentially making this a more favorable drug [13].

## SUPPORTIVE CLINICAL DATA

In addition to pharmacologic data, observational studies lend further support to the clinical efficacy of aminopenicillins in the treatment of ampicillin-resistant enterococcal UTIs. In a study of 20 male patients with ampicillin-resistant *Enterococcus* cystitis, all individuals treated with amoxicillin (vs nitrofurantoin) achieved symptom resolution [14]. Another observational study of 37 patients found no difference in clinical cure rates between those treated with aminopenicillins and those receiving alternative therapies for ampicillin-resistant infections [15]. A third study identified clinical cure of 88% across 84 hospitalized patients with ampicillin-resistant *Enterococcus* cystitis treated with ampicillin [16]. Finally, in a cohort of 178 patients (67% female) with vancomycin-resistant *Enterococcus* cystitis, clinical success rates were comparable at ∼80% between those treated with aminopenicillins and those managed with alternative agents [17]. Acknowledging the inherent limitations of observational studies, including the lack of long term follow up for recurrence of infections, the constellation of both pharmacokinetic-pharmacodynamic and clinical data are reassuring.

## RISK VERSUS BENEFIT CALCULATIONS

At the core of this discussion lies the recognition uncomplicated cystitis, while bothersome to patients, is generally not classified as a high-risk [18–20]. Uncomplicated cystitis carries a low likelihood of progressing to pyelonephritis or sepsis and, in many cases, resolves spontaneously. Indeed, clinical cure has been observed in 25–42% of women receiving placebo in randomized trials [21]. Given the typically benign course of uncomplicated cystitis, we contend that the risk-benefit calculus allows for a bolder approach—namely, the consideration of treatment strategies that might not be pursued in the context of higher-risk infections, such as those involving the upper urinary tract. Importantly, this more permissive therapeutic strategy is accompanied by several notable advantages: the use of narrower-spectrum agents, reduced potential for selective pressure and antimicrobial resistance, improved tolerability, minimized toxicity concerns, and enhanced affordability for patients.

## CONCLUSION

The CLSI defines resistance as when an organism's MIC exceeds achievable drug concentrations, when a recognized resistance mechanism is likely present, or when there is a lack of clinical evidence supporting therapeutic efficacy [1]. In adherence with this definition, we propose that the prevailing perspective on aminopenicillin-resistant *Enterococcus* warrants reconsideration. PK/PD data along with retrospective clinical analyses increasingly support the use of aminopenicillins for the management of uncomplicated cystitis caused by *Enterococcus* species, even when exhibiting ampicillin resistance. Acknowledging the lack of urine-specific breakpoints for *Enterococcus* and the unavailability of clinical trial data, we contend that aminopenicillins should not be categorically excluded as viable treatment options for cystitis due to ampicillin-resistant *Enterococcus*.
